# The unseen animal behind medicine: exploring considerations of animal-derived medications and anaesthetics in today's landscape

**DOI:** 10.1016/j.bjao.2024.100360

**Published:** 2024-12-16

**Authors:** Jennifer R. Wang, Eric Oh, Benjamin Aronow, Wendy K. Bernstein

**Affiliations:** Department of Anesthesiology, Boston Medical Center and Boston University Chobanian & Avedisian School of Medicine, Boston, MA, USA

**Keywords:** animal-derived medication selection, animal products, consent, ethics, non-animal alternatives, pharmaceutical innovation, religion, vegan

## Abstract

Requests for medical and anaesthetic care that is ‘vegan’ or free of animal-derived components are becoming increasingly common in the cultural landscape. Such requests are often rooted in religious beliefs and practices. There are currently no requirements for the disclosure of animal-derived components in medical items. However, both patients and medical professionals agree that greater transparency regarding such items is needed in obtaining informed consent. Although the ethical argument for disclosure has been established, there remain gaps in practical guidelines in recognising animal-derived components in medical items and understanding how to avoid them. This lack of comprehensive knowledge leads to challenges in initiating conversations about appropriate medication selection.

This manuscript will outline the common dietary restrictions of various religious groups and provide instruction on common animal-derived ingredients in medications. It will also introduce potential viable animal-free alternatives for some commonly used medications in the perioperative environment which has not been done previously in the literature. Moreover, we note the broader implications and reasoning behind considering dietary restrictions in medication choices.

The use of animal-derived components in various medical items, including medications, anaesthetics, and surgical implants, is gaining attention among patients with religious objections or dietary restrictions. These concerns are multifaceted, encompassing animal welfare considerations and potential conflicts with specific religious or dietary beliefs. There is currently a lack of mandatory disclosure regarding the use of animal-derived components in these products. However, studies indicate that a significant number of both patients and healthcare professionals consider informed consent critical in such situations.^1^, ^2^, ^3^, ^4^,^1^, ^2^, ^3^, ^4^ Many patients who observe dietary laws linked to their religious practices show a preference for alternatives to animal-based medications.^5^ Moreover, ethicists have argued that the ruling in UK's *Montgomery v Lanarkshire Health Board* case establishes a precedent requiring healthcare providers to disclose pertinent information about animal-derived components to patients, even if it means opting for a treatment that might be medically suboptimal.^2^^,^^6^

Improving awareness among healthcare professionals about religious and dietary restrictions related to animal-derived components in medications and anaesthetics is crucial for preventing the inadvertent prescription of non-compliant ingredients. There is currently a considerable gap in the training and resources on this subject for anaesthesia providers, leading to challenges in adequately informing patients who may have objections to certain medical agents. This oversight can inadvertently compromise patient autonomy and choice. By ensuring transparent disclosure of animal-derived ingredients, certain patient groups can be better equipped to make fully informed healthcare decisions.

This manuscript aims to outline the common dietary restrictions of various religious groups and to introduce potential viable animal-free alternatives for some commonly used medications in the perioperative environment. Additionally, we will explore the broader implications and reasoning behind considering dietary restrictions in medication choices, extending the discussion beyond the mere ethical concerns which have been covered in previous publications.

## Methods

A comprehensive review of existing literature on animal-free medications and anaesthesia options was conducted by using the electronic database PubMed. The PubMed keywords and search strings were as follows: ‘vegan anesthesia’ (PubMed Medical Subject Heading [MeSH]) AND ‘medication selection’ AND ‘vegetarian anesthesia’ AND ‘religion’ AND ‘vegan surgical implants’ AND ‘religious restrictions’ AND ‘porcine’ AND ‘bovine’ AND ‘animal-derived’ AND ‘animal-free alternatives’. Review articles, opinion pieces, case reports, and surveys of the attitudes of patients, practitioners, and religious leaders on the use of animal-derived medications were excluded. Eligible articles were scrutinised for practical guidelines on the identification of medications containing animal-derived components, and also suggestions on available animal-free alternatives. Because of the limited published literature on this topic, the authors also reviewed individual package inserts of various anaesthetic and critical care medications for potential animal-derived constituents. Data were extracted, reported, and then subsequently used to generate the proposed guidelines and substitutions in the tables.

## Results

We identified a total of 476 papers that fulfilled our criteria listed in the methods section. Although a number of papers have proposed the moral argument for the disclosure of animal-derived medications in obtaining patient consent, few have gone so far as to propose guidelines on how to identify such medications or how to choose substitutions.^1^^,^^2^^,^^4^^,^^7^, ^8^, ^9^, ^10^, ^11^, ^12^ Our literature review identified only a single paper that listed common medications containing animal-derived constitutes and possible substitutions, but this article does not address the implications of religious identity on medication selection.^13^

### Religion and dietary restrictions

In the USA, more than 18.1 million people identify with religious groups that may impact medication choices. The six most common groups are Judaism, Islam, Buddhism, Hinduism, Jehovah's Witnesses, and Seventh-day Adventists.^14^ Studies focusing on the impact of religious beliefs on medical decisions, particularly those regarding restricted ingredients, often defer to input by religious leaders, who may or may not represent an individual patient's beliefs.^3^^,^^9^^,^^10^^,^^15^ It is important to note that there is a wide range of practices within each religious group, including different sects within larger religious categories. Additionally, the level of adherence to religious dietary laws can vary greatly among individuals, making it risky to assume a patient's preferences based solely on their religious identity. For example, Jewish patients may or may not adhere to a kosher diet, leading to varying attitudes towards the use of porcine-derived elements in their medical care.

Recognising the complexity of patient attitudes towards animal-derived products in medicine calls for a nuanced and individualised approach in clinical discussions. Integrating these personalised conversations into preoperative consultations, medication management, and continuous care interactions is crucial for honouring patient autonomy. Physicians should be prepared to propose alternative treatments or work collaboratively with patients to find suitable options when direct substitutions are unavailable.

Table 1 serves as a concise summary of the dietary and medical restrictions associated with religious groups or sects within religious groups. It outlines specific restrictions that could impact medical treatment choices and describes the general stance of each religion on the use of restricted products in healthcare settings, including extenuating situations. This summary is useful for healthcare professionals as it provides a basic understanding for initiating informed discussions with patients. It aims to bridge the gap between standard medical practice and the diverse religious and ethical considerations of patients, thus aiding in a more personalised and respectful delivery of healthcare.

**Table 1:** Religious affiliations per US population with various restrictions.

Different religious sects	% of US population^14^	Restrictions that impact medical selection	Views on use of restricted products in medical situations
**Christian sects**			
Jehovah's Witness	0.8	• Blood and blood products are restricted.	Blood and blood products (platelets, white cells, unfractionated plasma, etc.) are forbidden. However, many Witnesses accept derivatives of primary blood products such as albumin, clotting factor concentrates, and immunoglobulins.^16^
Seventh-day Adventism	0.5	• Vegetarianism and veganism are common. • Alcohol restriction is common.	The use of animal-derived content in medical products is allowed.^17^
**Non-Christian religions**			
Buddhism	0.7	• Vegetarianism and veganism are common. • Alcohol restriction is common.	The use of animal-derived content in medical products is allowed.^15^ However, individual Buddhists may reject animal products based on their dietary preference.^9^
Hinduism	0.7	• Vegetarianism is common. • Bovine and porcine products are often restricted.	There is no universal stance on the use of bovine materials across Hinduism. Vaishnavism, the major branch of Hinduism, does not permit the use of any drugs, wound dressings, or implants if they contain bovine or porcine products. Religious exemptions may be made in emergencies if there are no available substitutions.^15^
Islam	0.9	• Vegetarianism is common. • Halal-observant diet prohibits porcine products, products from fish or shellfish, and alcohol.	Preferences vary, but religious exemptions are given for porcine products if no suitable substitute exists.^3^
Judaism	1.9	• Kosher diet prohibits porcine products, mixing of dairy with meat, meat that comes from non-cloven (split) hooves and do not eat grass, and fish that lack fins or scales.	Preferences vary, but porcine products are allowed if they are the safest treatment option.^18^

### Animal-derived excipients

Excipients comprise a variety of substances that are used as inactive ingredients in many pharmaceuticals, including tablets and capsules, which may be derived from animal sources. Because they are often not recognised as animal-derived, Table 2 is provided to give an overview of common animal-derived excipients with their sources and applications in the pharmaceutical industry.

**Table 2:** Common animal-derived excipients and their utility in pharmaceuticals.

Excipient	Source	Utility
Gelatine	Bovine or porcine bone, cartilage, or skin	Stabilising agent used in the production of hard and soft capsules, tablets, suppositories, emulsions, syrups, and dressings^19^
Glycerine	Animal or vegetable fat	Humectant and cosolvent used in oral solutions^20^
Lactose	Bovine (cow's milk)	Commonly used as a filler in oral tablets and capsules owing to its compressibility^21^
Magnesium stearate/stearic acid	Animal or vegetable fat	Magnesium stearate is derived from stearic acid, and is used as an emulsifier in producing medication tablets and capsules^22^
Shellac	Resin secreted from female *Kerria lacca* insects	Enhances shelf-life of tablet coatings^23^

Clear and standardised labelling on drug packaging, prominently highlighting the presence of animal-derived excipients through specific symbols or religious designations, could significantly reduce the risk of inadvertent consumption in those patients with dietary restrictions or ethical concerns. However, implementation of such changes in the USA would necessitate approval from the Food and Drug Administration (FDA). Currently, the FDA does not require labels to disclose whether a product contains animal-derived constituents or not.^24^

Healthcare professionals should be familiar with common animal-derived ingredients (i.e. gelatine or lactose) found in tablets and capsules and be prepared to offer alternatives. Options that are devoid of animal-derived medications, if commercially available, should be considered after careful deliberation.

### Animal-free alternatives for medications

There is currently a lack of centralised resources summarising which drugs contain animal-derived components. A list of pharmaceuticals derived from animals was released as part of the Australian Queensland Health Guidelines, but this resource was not intended to be used to screen for vegan medications and fails to disclose whether medications contain animal-derived excipients.^25^ Therefore, we generated a list (Table 3) as a practical guide to common perioperative medications and their recommended animal-free substitutions. This list may provide insight into the prevalence of animal-derived elements in medications. This table was created by screening individual drug packages through the US National Library of Medicine's DailyMed database for known animal-derived products.^4^ The authors are affiliated with a US institution; therefore, some drug agents mentioned here may not be available in all countries because of variations in regional pharmaceutical regulations and marketing approvals. Additionally, the variability in manufacturing practices may or may not lead to different formulations of the same drug containing the same animal-derived components.

**Table 3:** A summary of potentially problematic medications commonly used during operative procedures and in the ICU, with their associated animal-derived ingredients and suggested animal-free alternatives.^4^^,^^13^^,^^25^, ^26^, ^27^, ^28^ ∗I.V. forms of medications can impact access, potency, and expense, as they require patients to have safe and reliable venous access and monitored care. Note that one of the most serious complications of venous access is infection.

Drug	Ingredient/Source	Animal-free alternative	Potential issues with alternative
**Analgesics**			
Acetaminophen/Paracetamol p.o.	Regular strength: magnesium stearate Extra strength: magnesium stearate, shellac	I.V. form∗	
Hydrocodone/acetaminophen p.o.	Tablets: magnesium stearate Oral solution: Glycerine	None available	
Lidocaine patch	Gelatine (bovine)	5% Lidocaine ointment (polyethylene glycol-based formulations only)	
Oxycodone p.o.	Tablets: lactose, magnesium stearate	Oral solution	
Tapentadol	Lactose	None available	
Oxycodone/naloxone p.o.	Lactose	None available	
Tramadol p.o.	Tablets: magnesium stearate Oral solution: Glycerine	Injectable solution	
**Anaesthetics and sedatives**			
Alprazolam p.o.	Lactose, magnesium stearate	None available	
Diazepam p.o.	Lactose, calcium stearate (plant or animal source)	I.V. form∗, oral solution (diazepam intensol), p.r.	
Propofol	Avian (egg lecithin from purified chicken egg yolks)	Propofol (Cleofol®), fospropofol	Propofol (Cleofol®): excessive pain on injection^29^ Fospropofol: slower onset of action than propofol.^30^ Care must be taken in supplemental dosing to avoid oversedation.^31^ Transient paraesthesia and pruritus are common.^32^
Lorazepam p.o.	Lactose, magnesium stearate	I.V. form∗	
Temazepam p.o.	Gelatine, magnesium stearate, lactose, shellac	Midazolam (i.v. or buccal)	
**Anticoagulants**			
Abciximab	Murine	None available	
Eptacog alfa (recombinant factor VIIa)	Bovine, hamster, murine	None available	
Factors II, V, VII, IX, X	Porcine	None available	
Heparin	Bovine and porcine	Argatroban, Bivalirudin [4]	Can increase risk of bleeding as it lacks a specific antagonist^26^
Heparin, low molecular weight	Porcine	Fondaparinux (Arixtra®)^33^	
Heparinoids–danaparoid	Porcine	None available	
Hirudin drugs	*Hirudo medicinalis* leech	None available	
**Antiemetics**			
Aprepitant p.o.	Gelatine	I.V. form∗	
Metoclopramide p.o.	Lactose, stearic acid	I.V. form∗	
Ondansetron p.o.	Tablet: lactose, magnesium stearate Oral disintegrating tablet: gelatine from porcine or bovine sources	Oral solution, i.v. form∗	
**Hormones**			
Oestrogen, conjugated	Equine	Oestradiol topical gel, vaginal ring	
Corticotropin	Porcine	None available	
Non-human insulins	Various sources (bovine, porcine, fish)	Recombinant insulins	
**Miscellaneous**			
Butylscopolamine p.o.	White beeswax	Topical form	
Clonidine p.o.	Lactose, magnesium stearate	I.V. form∗	
Diphenhydramine p.o.	Magnesium stearate	I.V. form∗	
Gabapentin	Capsule: gelatine (bovine and porcine), shellac Tablet: magnesium stearate Oral solution: glycerine	I.V. form∗	
Gelofusine	Gelatine (bovine and porcine)	Other colloids	
Intralipid	Avian (purified egg phospholipids)	None available	
Oxybutynin p.o.	Lactose, magnesium stearate	None available	
Pregabalin	Lactose, gelatine (unspecified), shellac	Oral solution	
Protamine sulfate	Salmon sperm (original source)	None available	
Seretide	Lactose	Evohaler	
Tenecteplase	Hamster	Alteplase	Slightly inferior performance^34^

It is worth noting that although the proposed substitutions in this manuscript are primarily aimed at addressing ethical and cultural concerns, they are also well-suited for patients with specific allergies to animal-derived products. As such, this table provides additional versatile applications for healthcare professionals to consider.

#### Propofol

The lack of an animal-free anaesthetic induction agent has been a longstanding challenge for anaesthesiologists in some countries.^2^^,^^7^^,^^13^ Propofol, the most commonly used induction agent in anaesthesia, contains egg lecithin (lipids from purified egg yolks), making it unsuitable for some vegan patients. An alternative, Cleofol®, which is egg-free, is commercially available from a manufacturer in India; however, it is associated with even greater pain on injection than standard formulations of propofol.^29^

Fospropofol has been released as an alternative to liquid emulsion propofol. As a water-soluble prodrug of propofol, fospropofol first requires metabolism into propofol, resulting in a slower onset of action (4–13 min) than propofol's rapid effect (40 s).^30^^,^^35^^,^^36^ Care must be taken to avoid oversedation, as supplemental doses of fospropofol should not be administered more frequently than every 4 min.^31^ Fospropofol has also been associated with transient paraesthesia and pruritus, probably caused by an ester present in the drug formulation, but offers advantages such as less injection pain and better shelf stability.^32^

Thiopental has been recommended as an acceptable animal-free alternative to propofol because of its similar performance profile.^13^^,^^37^^,^^38^ However, the production of thiopental ceased in the USA in 2021 while other jurisdictions, such as the European Union, banned its export because of its use in capital punishment, leading to a shortage and subsequent unavailability in the USA^39^ and withdrawal from use in many European Union countries. The US decision was contested by the American Society of Anesthesiologists, which argued for its utility in specific anaesthetic contexts, especially where propofol is not advisable.^40^ Thiopental is still listed in the British National Formulary and in the current WHO list of essential medicines (as an alternative to propofol).^41^

Other vegan-friendly induction agents, such as ketamine and etomidate, are available but associated with poorer outcomes in critically ill patients.^42^ They have not been listed as alternatives to propofol in Table 3 because of their distinct application profiles and safety considerations when compared with propofol, although they may be appropriate in specific clinical situations.

## Discussion

The ethical imperative for informing patients about animal-derived components in their medications and anaesthetics aligns with Beauchamp and Childress' four principles approach, emphasising the necessity of disclosure for maintaining patient autonomy, non-maleficence, beneficence, and justice.^2^ Furthermore, the development of cultural awareness and competency should be an on-going process of patient-centred care. Healthcare providers are encouraged to familiarise themselves with animal-derived medications and anaesthetics to facilitate informed consent effectively.

Although the argument for disclosing the presence of animal-derived components often centres on respecting the patient's religious identity and dietary preferences, there is an equally important consideration for patient safety. Dietary restrictions are sometimes imposed because of allergies or hypersensitivity reactions to specific components. Although rare, documented cases exist where inadvertent administration of animal-derived medications caused hypersensitivity reactions.^43^ For instance, when lactose-containing medications are contaminated with milk protein, they can cause allergic reactions, including anaphylaxis, in patients with cow milk allergy.^44^^,^^45^ There have also been reports of adverse reactions such as pruritus, urticaria, and even cardiogenic shock in patients treated with animal protein-derived heparin.^46^^,^^47^ Furthermore, practitioners are advised to exercise caution with NovoSeven® (recombinant factor VIIa) in patients with known hypersensitivities to mouse, hamster, or bovine proteins.^48^

The critical point of the issue is the absence of centralised resources that consolidate these risks for healthcare providers. There is no database of medications containing animal-derived elements, nor is there clear labelling indicating if a medication is vegan. Current practice requires physicians to recognise animal-derived components in medications and to screen for them in individual drug package inserts. As it stands, the process is time-consuming, burdensome, and impractical to complete for every medication given in the perioperative period. Given these challenges, the authors recommend that physicians proactively inquire about a patient's dietary preferences during consultation and educate themselves on how these preferences could influence medication choices. This ethical approach supports transparency between provider and patient. It also respects patient autonomy and enhances patient safety by reducing the risk of adverse reactions.

Yet this approach must be balanced against placing an unacceptable burden on both the anaesthesia provider and the patient. Reviewing and discussing the animal content of medications with every patient can be incredibly time-consuming. In addition, there are potential trade-offs with the substitution of alternative drugs and therapies which can be equally complex and burdensome. This does not suggest that anaesthesia personnel who do not raise these issues with every patient is neglectful of their duties. Rather, that raising such conversations regarding dietary preferences and animal content of every medication should be balanced against the risk of placing an unacceptable burden on either the caregiver or patient.

In acknowledging the availability of substitutes for medications containing animal-derived ingredients, it is important to recognise the mere existence of an alternative does not guarantee its immediate or widespread availability. Hospital pharmacies might not always have these substitutes in stock, presenting a practical limitation for healthcare professionals and patients alike. Additionally, opting for an animal-free alternative could inadvertently lead to increased healthcare barriers. For instance, a patient preferring to avoid gelatine in capsules might opt for an i.v. formulation. This preference necessitates i.v. access, potentially increasing the complexity, cost of treatment, and risk for infection. Such factors must be carefully weighed during patient consultations. In addition, with regard to ‘vegan’ anaesthesia, there are seldom ‘real’ alternatives available. Available alternative medications are often not realistic options, scarcely available, produce more side-effects, or unnecessarily complicate a patient's treatment plan.

One should also explore the use of laboratory animals in the production and testing of anaesthetic drugs, which has long been a standard practice. As awareness grows about animal welfare and the need to reduce reliance on animal testing, the development of alternative non-animal stabilising agents in medications is becoming increasingly important. Advancing research in this area is crucial to align medical practices with evolving ethical standards and to enhance the overall quality of patient care.

Healthcare providers should discuss these logistics and financial challenges openly with patients, ensuring that their medical decisions are made with a full understanding of all possible implications. This approach helps maintain a balance between respecting patient preferences and practical treatment considerations.

This review has a few limitations. Firstly, the search had its challenges, as there is a paucity of literature on this subject. Many manuscripts focused on the ethical considerations of informed consent in patients with dietary restrictions rather than the practical applications of the process. Furthermore, there was no high-quality, comprehensive database available which lists animal-derived components within medications and anaesthetics. As the authors are affiliated with a US institution, we acknowledge that the pharmaceutical agents listed may not be as applicable to practices outside the USA. We opted for broad inclusion of medications which often contain animal-derived ingredients to build a set of practical guidelines. However, this may lead to factual inaccuracies owing to differences in the manufacturing process of pharmaceuticals. As such, not every listed medication will contain animal-derived components unless specifically disclosed by the manufacturer.

### Conclusions

Enhancing healthcare professionals' understanding of the presence of animal-derived components in medications and integrating routine inquiries about patient's religious and dietary preferences into clinical practice are crucial steps towards empowering patients in their medical care decisions. This approach fosters a more inclusive and respectful healthcare environment, where patient autonomy is prioritised.

To achieve this, comprehensive education for healthcare providers is essential, focusing on the identification of animal-derived ingredients, their possible animal-free substitutions, and their potential conflicts with various religious beliefs and dietary restrictions. By engaging patients in detailed discussions about their personal preferences and values, healthcare providers can facilitate more ethically informed medication choices. Such dialogues are vital in ensuring that a patient's consent is truly informed and respects their individual beliefs and needs.

Furthermore, when animal-free alternatives are not available, it becomes imperative for healthcare providers and patients to collaboratively explore potential substitutions. These compromises should aim to balance the therapeutic efficacy of treatment with the patient's personal values and beliefs. This collaborative approach not only enhances patient satisfaction and trust in the healthcare system but also ensures that care is delivered in a manner that is both clinically effective and ethically sound.

Looking to the future, there is a need for the development of more animal-free medical products and clearer labelling practices, to better accommodate the diverse needs of patients. The healthcare industry should strive to address these gaps, ensuring that patient care is not only effective but also aligns with the evolving ethical landscape of medicine and values patient autonomy. This progressive shift will not only improve patient outcomes but also reflect a broader commitment to inclusivity and respect for individual choices in healthcare.

## Author’s contributions

Conceptualisation: all authors

Research: JW, EO, WB

Manuscript writing: JW, EO, WB

Manuscript editing: JW, WB

## Declarations of interest

The authors declare that they have no conflicts of interest.
